# Methyl gallate, gallic acid-derived compound, inhibit cell proliferation through increasing ROS production and apoptosis in hepatocellular carcinoma cells

**DOI:** 10.1371/journal.pone.0248521

**Published:** 2021-03-16

**Authors:** Chien-Yu Huang, Yu-Jia Chang, Po-Li Wei, Chin-Sheng Hung, Weu Wang

**Affiliations:** 1 Department of Surgery, School of Medicine, College of Medicine, Taipei Medical University, Taipei, Taiwan; 2 Division of General Surgery, Department of Surgery, Shuang Ho Hospital, Taipei Medical University, Taipei, Taiwan; 3 Division of Colorectal Surgery, Department of Surgery, Shuang Ho Hospital, Taipei Medical University, Taipei, Taiwan; 4 Department of Pathology, Wan Fang Hospital, Taipei Medical University, Taipei, Taiwan; 5 Graduate Institute of Clinical Medicine, College of Medicine, Taipei Medical University, Taipei, Taiwan; 6 Cell Physiology and Molecular Image Research Center, Wan Fang Hospital, Taipei Medical University, Taipei, Taiwan; 7 Division of Colorectal Surgery, Department of Surgery, Taipei Medical University Hospital, Taipei Medical University, Taipei, Taiwan; 8 Cancer Research Center and Translational Laboratory, Taipei Medical University Hospital, Taipei Medical University, Taipei, Taiwan; 9 Graduate Institute of Cancer Biology and Drug Discovery, Taipei Medical University, Taipei, Taiwan; Columbia University, UNITED STATES

## Abstract

Hepatocellular carcinoma (HCC) is a global health problem. Currently, there is no effective therapeutic strategy for HCC. Methyl gallate (MG), from plant-derived phenolic gallic acid, has exhibited antitumor efficacy. However, the effect of MG on HCC is unclear. In vitro growth activity was detected by a sulforhodamine assay. A zebrafish xenotransplantation was applied to evaluate the inhibitory effect of MG. Reactive oxygen species (ROS) production, autophagy, and lysosome formation were detected by specific dyes. Finally, apoptosis was examined using annexin V-FITC/PI staining and western blot was performed to determine the molecular mechanism. It was demonstrated that MG treatment inhibited the proliferation of Hep3B, Mahlavu, and HepJ5 cells. Xenotransplantation also showed that MG inhibited the growth of Hep3B and HepJ5 cells. MG treatment increased cellular levels of superoxide and oxidative stress. Increases in autophagy and lysosome formation were found after MG treatment. The western blot analysis showed that MG activated cleavage of caspase-3 and poly (SDP ribose) polymerase (PARP), modulated levels of the Bcl2, Bax, and Bad ligands, and induced apoptosis. MG induced autophagy with notable activation of beclin-1, autophagy related 5+12 (ATG5+12), and conversion of light chain 3-I (LC3-I) to II. Our study showed that MG exposure inhibited HCC proliferation both in vitro and in vivo. And blocking autophagy enhanced MG-induced cytotoxicity in HCC cells. These findings suggested MG might serve as a powerful therapeutic supplement for human HCC patients.

## 1. Introduction

Hepatocellular carcinoma (HCC) is a highly prevalent cause of death worldwide [1, 2]. Fewer than 30% of newly diagnosed patients tolerate curative surgical treatment or liver transplantation [3, 4]. The poor prognosis of HCC is related to the high recurrence or metastasis rate after surgical treatment [5–7]. New approaches for preventing, diagnosing, and treating HCC urgently need to be developed. In HCC, there is a progressive linking of chronic inflammation with cirrhosis and carcinogenesis. Accumulating evidence has revealed that inflammation is related to invasion and metastasis of human cancers [8]. It is important to clarify the mechanisms of the carcinogenesis and metastasis of HCC and identify effective therapeutic agents [9].

Radiotherapy and chemotherapy for HCC treatment produce significant complications with limited responses. Recent attention has focused on seeking safe and effective agents from natural remedies for chemoprevention, especially from traditional Chinese medicine [10]. Methyl gallate (MG), methyl-3,4,5-trihydroxybenzoic acid, is prevalent phenolic compounds in plants [11]. It was reported MG decreased oxidative stress and DNA damage related to hydrogen peroxide in MDCK cells. And MG, similar to vitamin E analogues, reduced lipid peroxidation and prevented depletion of intracellular glutathione (GSH) [12]. Moreover, MG showed low cytotoxic effects against the HaCaT normal skin cell line [13]. Thus, although MG is generally recognized as safe, it possesses antioxidant abilities and inhibits lipid peroxidation [14].

Aside from its antioxidant activity, MG also exhibits multiple biological properties that include anti-spasmodic, anti-atherogenic, anti-inflammatory, and anti-microbial activities [12, 15–17]. MG was reported to have a protective effect against oxidative stress in erythrocytes [18], adipocytes [14], vascular endothelial cells [19], cardiac myocytes [20], and brain and neural networks [21, 22]. MG inhibited focal adhesion formation, and reduced cell viability and migration in glioma cells. Mechanically, downregulation of the protein kinase B (AKT)/phosphorylated AKT and extracellular signal-regulated kinase (ERK) signaling pathways was noted [23, 24]. MG modulated immune responses by inhibiting interleukin (IL)-6 and IL-8 in human oral epithelium cells [16]. Cancer-bearing hosts often exhibit detectable specific immunity against tumor-associated antigens. MG modulated antitumor immunity in lymphomas by inhibiting tumor infiltration of CD4+ CD25+ T-regulatory cells (Tregs) and showed a synergic effect with cisplatin [25, 26]. However, information on oxidative stress and biological activities related to MG in HCC cells is scanty.

Oxidative stress is constantly generated by aerobic metabolism and includes peroxides and free radicals [27, 28]. Oxidative stress causes cell damage, as well as DNA strand breaks [29, 30]. Redox homeostasis is mediated via the balance between ROS production and antioxidant scavengers [31]. A hypoxic microenvironment is common in rapidly growing solid tumors, like HCC [32]. Aerobic metabolism is very important for cancer cells to adapt to a hypoxic status and reach a steady-state over time, and it even induces more-aggressive/resistant cells [27, 33]. Various protective mechanisms against oxidative stress have been identified, including the transient overproduction of ROS inside cells. Large amounts of ROS or reactive nitrogen species (RNS) production can change expression patterns and signal pathways, which in turn may induce cell death or give rise to carcinogenesis. MG and N-acetylcysteine were reported to exhibit protective efficacy in rat pheochromocytoma cells by decreasing H_2_O_2_-induced apoptosis [34]. It was suggested that antioxidative and cytoprotective properties of MG may change to pro-oxidative and cytotoxic properties in different cell lines or microenvironments [35].

While MG has a proven inhibitory effect on glioma, lymphoma, and human epidermoid carcinoma cells, its general antitumor effects and the detailed mechanism of how it regulates apoptosis and/or autophagy in human HCC cells remain unknown. Herein, we attempted to explore the biological roles and redox signaling of MG in HCC.

## 2. Materials and methods

### 2.1. Chemicals, reagents, and cell culture

All the chemicals used in the study were purchased from Sigma (St. Louis, MO, USA). Antibodies targeting ATG5/12, Beclin-1, LC3, GAPDH, Bcl-2, Bax and cleaved caspase3 (C-caspase3) were obtained from Cell Signaling Technology (Danvers, MA, USA), and antibodies for cleaved poly (ADP ribose) polymerase (c-PARP) were purchased from Santa Cruz Biotechnology (Santa Cruz, CA, USA). Hep3B and Mahlavu cell lines were purchased from American Type Culture Collection (ATCC, Manassas, VA, USA), and HepJ5 cells were established by Dr. C. S. Yang’s laborotary as previously described [36]. HCC cell lines (Hep3B, HepJ5, and Mahlavu) were grown and maintained in Dulbecco’s modified Eagle’s medium (Life Technologies, Grand Island, NY, USA) supplemented with 10% (v/v) fetal calf serum in a 5% CO_2_ humidified incubator at 37°C.

### 2.2. Sulforhodamine B (SRB) colorimetric assay for cytotoxicity screening

Initially, 2×10^4^ cells were seeded in each well of 24-well plates. After overnight incubation in the CO_2_ incubator, different doses of MG (0~40 μg/ml) were added into the wells and leave for 24 or 48 h. Next, the treated cells were fixed with 10% trichloroacetic acid overnight and then stained with protein-bound SRB for 30 min. After staining, cells were washed twice with 1% acetic acid to remove excess dye. A 10 mM Tris base solution was used to dissolve the protein-bound dye. The optical density was measured with a microplate reader at 515 nm (Bio-Rad Laboratories, Hercules, CA, USA).

### 2.3. Xenotransplantation assay

The xenotransplantation process was performed at the Taiwan Zebrafish Core Facility-Human Disease Model Resource Center. Briefly, zebrafish embryos of 2 days post-fertilization (dpf) were dechorionated and anesthetized with tricaine (0.04 mg/ml; Sigma). The HCC cells, HepJ5 or Hep3B, were detached and collected from the culture wells for CM-Dil (red fluorescence) (Vybrant; Invitrogen, Carlsbad, CA, USA) labeling. The labeled cells (4.6 nl, approximately 200 cells) were injected into the yolk of each 2-dpf embryo using a Nanoject II Auto-Nanoliter Injector (Drummond Scientific, Broomall, PA, USA). After implantation, the zebrafish embryos (n = 20 for each group) were washed with fish water once to remove the residual chemicals and incubated at 28°C for 1 h. Later, either distilled H_2_O or MG at doses of 0, 40μg/ml were applied to the embryos. Fluorescent cells in the embryos were checked at 2 h post-implantation and examined at 1 and 3 days post-injection (dpi) by fluorescence microscopy.

### 2.4. Total ROS/superoxide detection using the FlexiCyte^TM^ protocol

The intracellular ROS were measured using a total ROS/Superoxide Detection Kit (Enzo Life Science, Farmingdale, NY, USA) following the manufacturer’s instructions. In brief, cells were stained with the two-color ROS Detection Kit and monitored using the NucleoCounter^®^ NC-3000^TM^ system (ChemoMetec, Allerod, Denmark). The detail protocol was modified from the one previously described [37].

### 2.5. Autophagy detection using an autophagy detection kit

CYTO-ID® Autophagy Detection Kit (ENZ-51031, Enzo) was used to measure autophagic activity according to the manufacturer’s instructions. 2.4×10^5^ cells were seeded into each well of six-well plates and grown in a CO_2_ incubator at 37°C overnight. The next day, MG or vehicle was applied to the cells for 24 h. Number of autophagic vacuoles was measured and the autophgic flux was monitored after the cells were harvested and stained with fluorescent dyes. The fluorescence intensity and number of autophagosomes were detected and measured using the NucleoCounter^®^ NC-3000^TM^ system (ChemoMetec) [37].

### 2.6. Lysosome formation

Lysosome formation induced by MG was measured using the LYSO-ID® Green Detection Kit (ENZ-51034, Enzo). 2.4×10^5^ cells were seeded into each well of six-well plates and cultured in a CO_2_ incubator at 37°C overnight. MG or the vehicle were used to treat the cells for 24 h, and the cells were harvested and stained with fluorescent dyes using the LYSO-ID® Green Detection Kit as described by the manufacturer’s. Fluorescence intensity was measured using the NucleoCounter® NC-3000^TM^ system (ChemoMetec) [37].

### 2.7. Protein extraction and western blot analysis

Proteins were extracted from the cells treated with MG or the vehicle for 48 h and were analyzed by western blotting as previously described [38]. Briefly, aliquots of total 20 μg proteins were denatured and separated by sodium dodecylsulfate polyacrylamide gel electrophoresis (SDS-PAGE) followed by electrotransfering onto polyvinylidene difluoride membranes (GE Healthcare Piscataway, NJ, USA). The membranes were incubated with BSA for 1h and then incubated with ATG5/12, Beclin-1, LC3, Bcl2, Bax, c-PARP or c-caspase3 primary antibodies overnight at 4°C individually. The respective secondary antibodies were subsequently probed, and the signals were amplified by using an enhanced chemiluminescence reagent (GE Healthcare) and visualized using VersaDoc 5000 (Bio-Rad Laboratories, Hercules, CA, USA).

### 2.8. Annexin V-FITC/ Propidium Iodide (PI) double staining assay

Annexin V-FITC Apoptosis Detection Kit (Cat No.: AVK250) was purchased from Strong Biotech Corporation. Cells were cultured in 6-well plates at cell density of 2.4×10^5^ cells /well and treated with MG (40 μg/ml) for 48h alone or in combination with CQ (10 μM) for 16 h. After treatment, the medium was removed and the cells were centrifuged and washed with PBS. After the supernatant was discarded, the cell pellets were resuspended in 100 μL of binding buffer. Then Annexin V and PI working solution were added to cell suspension and incubated at room temperature for 15 minutes. Then the samples were analyzed using a flow cytometer (BD Biosciences, San Diego, CA, USA).

### 2.9. Statistical analyses

Statistical analysis was performed using statistics functions of Microsoft Excel. Data are presented as mean±standard deviation (SD) of at least three independent experiments. For IC_50_ experiments, statistical significant differences were used one-way ANOVA or Student’s *t*-test (two-tailed) (*, p<0.05; **, p<0.01).

## 3. Results

### 3.1. MG inhibits the growth of HCC cells

**Fig 1:**
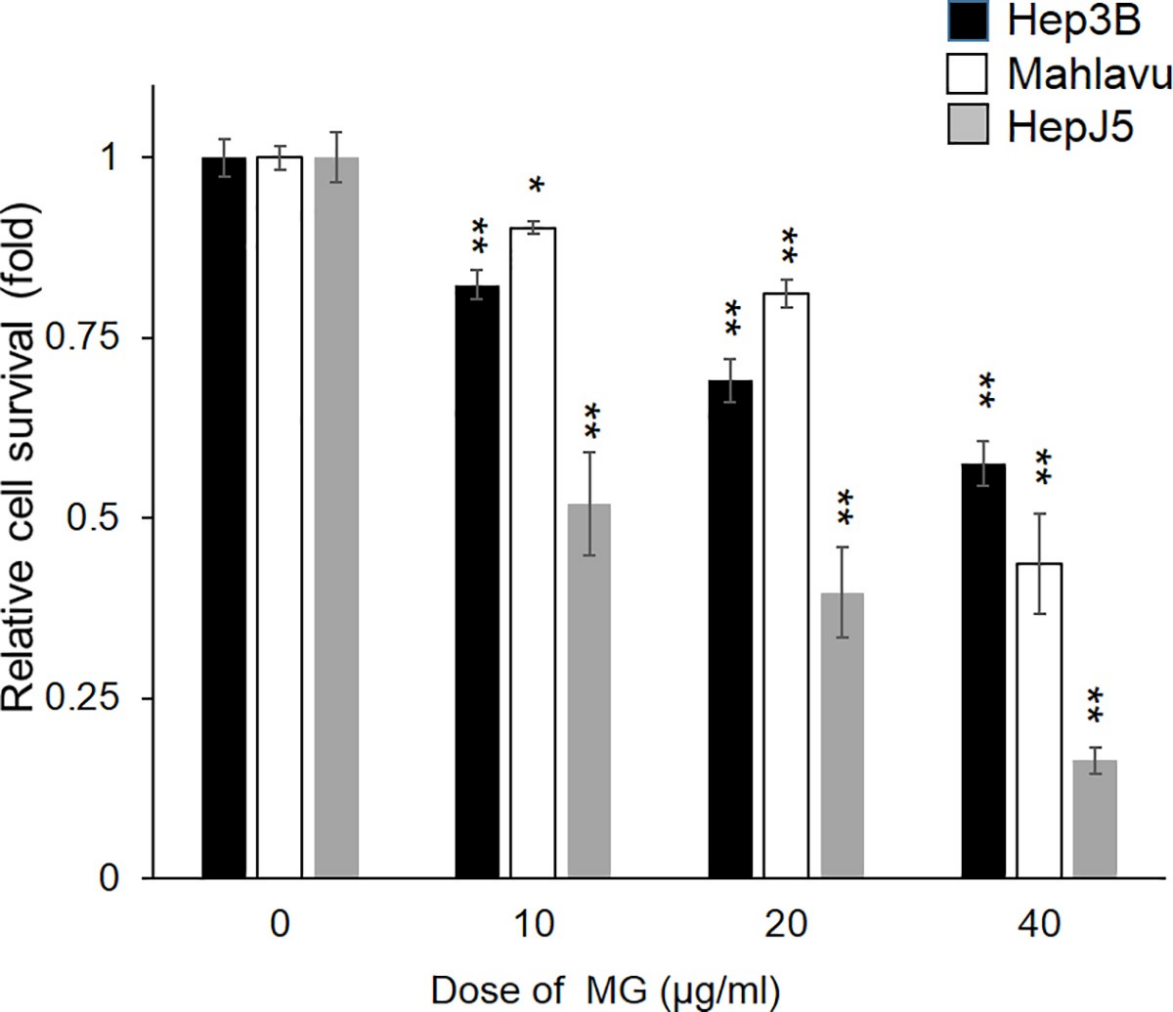
Methyl Gallate (MG) treatment decreases hepatocellular carcinoma (HCC) cell survival. After treating Hep3B, Mahlavu, and HepJ5 cells with different doses of MG (0~40 μg/ml) for 48h, the relative cell survival rate was determined using an SRB assay. Survival of vehicle-treated cells was defined as 100%. MG treatment showed reduction of the cell survival rate in HCC cells in a dose-dependent manner. Data are expressed as the mean±SD of three independent experiments in triplicate (* p<0.05, ** *p*<0.01).

First, we investigated the cytotoxic effects of MG on HCC (Hep3B, Mahlavu, and HepJ5) cells by an SRB assay. As shown in Fig 1, MG treatment markedly decreased the proliferation of Hep3B, HepJ5, and Mahlavu cells in a dose-dependent manner (Fig 1). The 50% inhibitory concentration values of MG were >40, ~40, and ~20 μg/ml for Hep3B, Mahlavu, and HepJ5 cells at 48h, respectively. These data indicate that MG treatment inhibits significantly the cell survival of Hep3B, HepJ5, and Mahlavu cells.

### 3.2. MG suppressed HCC cell proliferation in a zebrafish model

**Fig 2:**
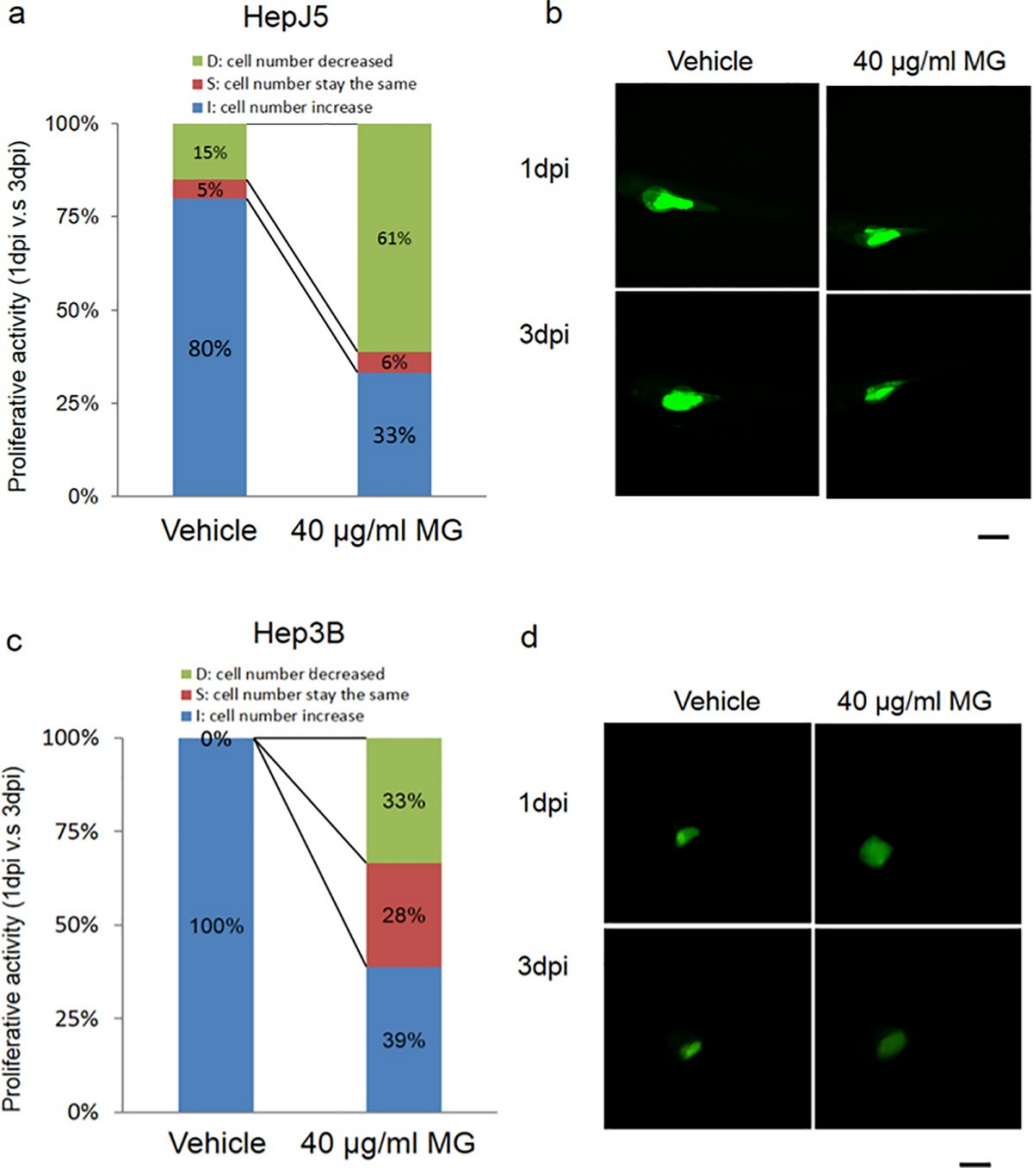
MG-suppressed cell proliferation in a xenotransplantation model. Zebrafish was used as the animal model for the xenotransplantation assay to determine the efficacy of MG treatment in hepatocellular carcinoma (HCC). Fluorescence labeled Hep3B and HepJ5 cells were implanted into an embryo yolk of the zebrafish, and then embryos were exposed to 40 μg/ml MG or dH_2_O as a vehicle control. Proliferative activities of the HCC cell lines in the embryos (n = 20 for each group) were compared by monitoring the fluorescence intensity on days 1 and 3 post-injection (1 and 3 dpi) of MG. (a and b) MG treatment reduced the increase in cell numbers in the embryo population (from 80% for the vehicle to 33% embryos respectively) in HepJ5 cells. A decrease in the fluorescence intensity was shown after 3 days in Hep3B cells with 40 μg/ml MG treatment (c and d). In the Hep3B cell line, the increase in cell numbers in the embryo population decreased from 100% (vehicle) to 39% (40 μg/ml) within 20 embryos. Treatment with 40 μg/ml MG dramatically decreased the fluorescence intensity in HCC cells compared to the vehicle. Scale bare was 1 mm.

We used a zebrafish xenotransplantation assay to further evaluate the effect of MG treatment on HCC. As shown in Fig 2, Hep3B and HepJ5 cells were stained by the carboxyfluorescein succinimidyl ester (CFSE) florescence dye and then implanted into a zebrafish embryo yolk. The florescence intensity was monitored at 1 dpi, and after being treated with the drug for 2 days, was monitored at 3 dpi. We compared 1- vs. 3-dpi stages to demonstrate the proliferative activity between HepJ5 or Hep3B cells treated with vehicle, or 40 μg/ml MG. Numbers of HepJ5 cells were dramatically reduced in MG-treated embryos compared to vehicle-treated embryos (Fig 2A and 2B). The same trend was found in Hep3B-injected embryos (Fig 2C and 2D). Numbers of increased cells in MG-treated embryos were lower than those in vehicle-treated embryos (100% vs. 39%). Our results indicated that MG treatment caused a decrease in the cell growth ability of HepJ5 and Hep3B cells.

### 3.3. MG enhances ROS and superoxide generation

**Fig 3:**
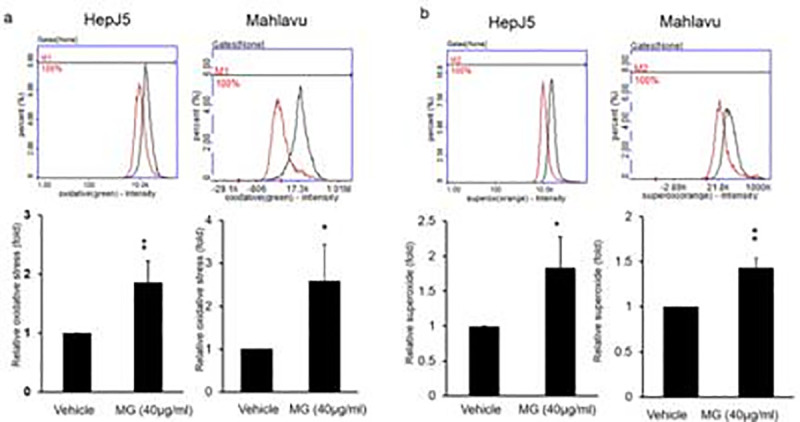
Methyl Gallate (MG) increases Reactive Oxygen Species (ROS) and superoxide levels in HepJ5 and Mahlavu cells. Both of HepJ5 and Mahlavu cells were treated with 40 μg/ml MG for 24 h. ROS and superoxide levels were detected using specific dyes. MG treatment significantly increased ROS and superoxide levels compared to the vehicle. Data are presented as the mean±SD of three independent experiments in triplicate (* *p*<0.05, ** *p*<0.01).

**Fig 4:**
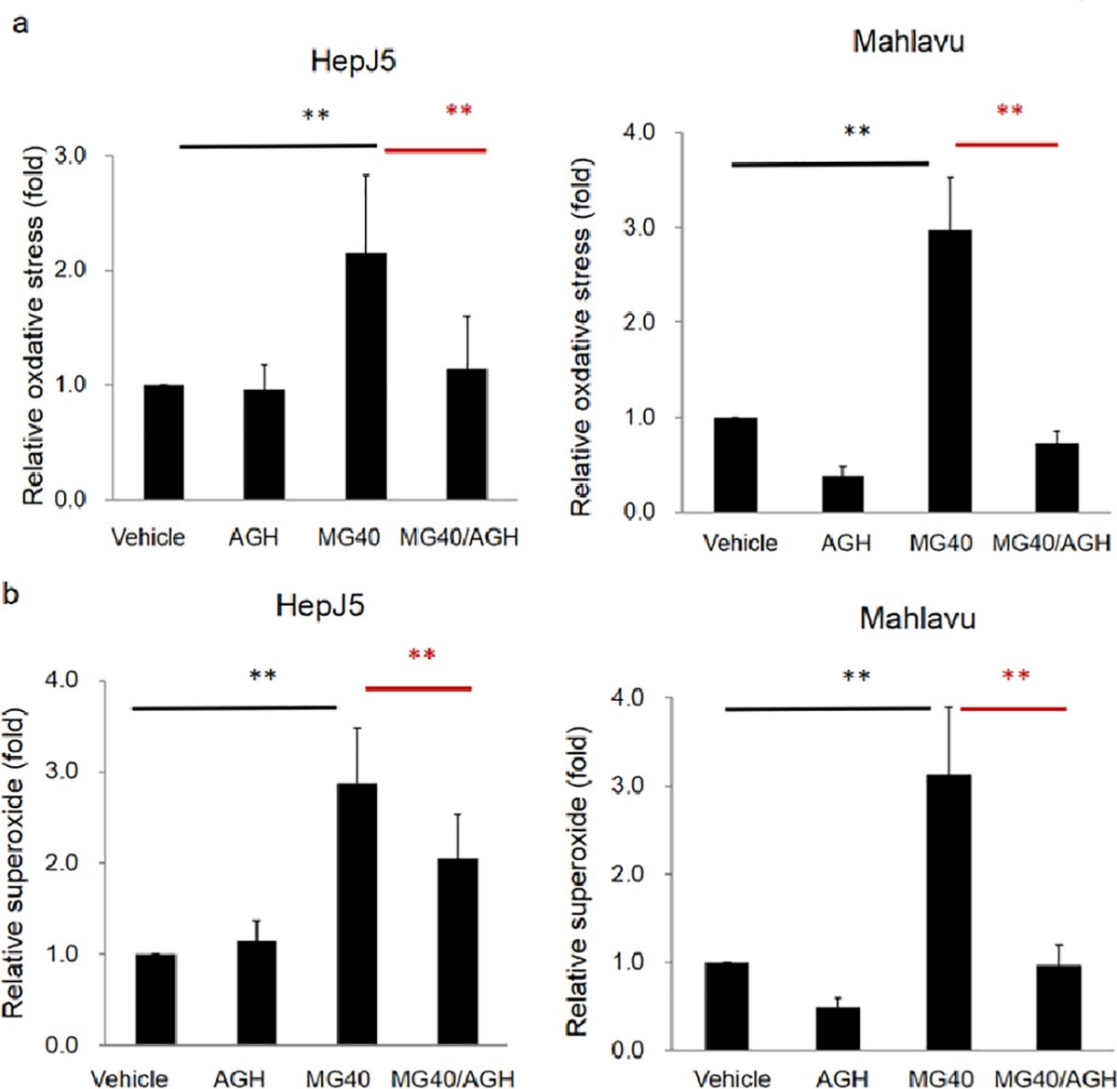
Pretreatment with aminoguanidine hemisulfate (AGH; an antioxidant) abolishes the Methyl Gallate (MG)-induced reactive oxygen species pretreatment with aminoguanidine hemisulfate (AGH; an antioxidant) abolishes the Methyl Gallate (MG)-induced Reactive Oxygen Species (ROS) and superoxide levels. HepJ5 and Mahlavu cells were treated with AGH and then exposed to MG. Levels of ROS and superoxide were detected using specific fluorescence dyes. (a) Significant increase of the ROS level was found after MG treatment. The MG-induced ROS level was abolished in AGH pretreated HepJ5 and Mahlavu cells. (b) The superoxide level increased after exposure to MG and was abolished with AGH pretreatment. Data are presented as the mean±SD of three independent experiments in triplicate (** *p*<0.01).

ROS production is involved in apoptosis and/or autophagy as documented in several previous reports. We detected ROS levels in MG-treated HepJ5 and Mahlavu cells using an ROS/Superoxide Detection Kit. As shown in Fig 3, exposure of HepJ5 and Mahlavu cells to 40 μg/ml MG for 24 h significantly increased intracellular oxidative stress and superoxide production (Fig 3). Levels of ROS generation and superoxide generation in HepJ5 reached 1.9- and 1.8-fold, respectively after MG exposure (Fig 3A and 3B). The similar results were found in Mahlavu cells. These results showed that MG induced intracellular ROS levels and superoxide generation in HCC cells. We examined the effects of aminoguanidine hemisulfate (AGH) on ROS and superoxide levels in MG-treated HCC cells. AGH, a well-known antioxidant, is a diamine oxidase and nitric oxide synthase inhibitor [39]. AGH significantly blocked ROS and superoxide levels in MG-treated HepJ5 and Mahlavu cells (Fig 4A and 4B). These results indicated that MG exposure can induce ROS production, and pretreatment with an antioxidant can suppress MG-induced ROS production.

### 3.4. MG induces autophagy and lysosome formation in HCC cells

**Fig 5:**
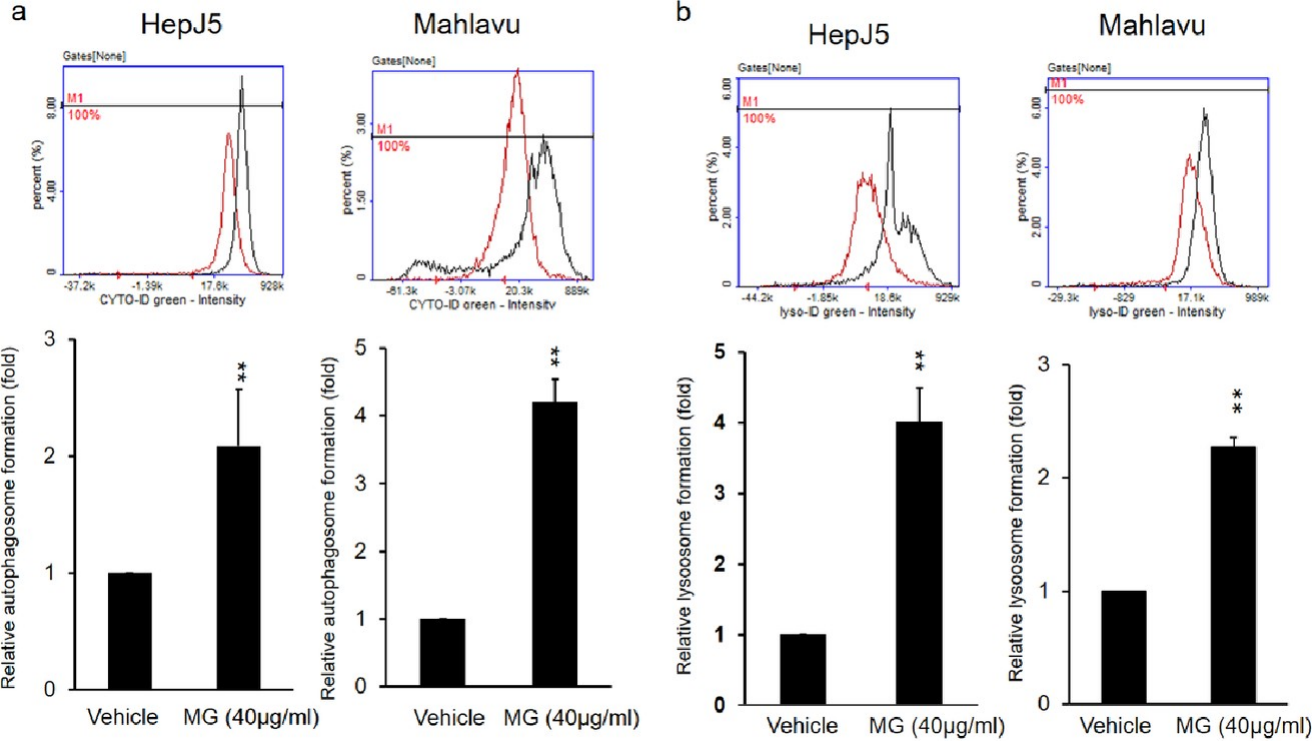
Methyl Gallate (MG) increases autophagosomes and lysosomes formation. HepJ5 and Mahlavu cells were exposed to 40 μg/ml MG for 24 h. (a) Autophagosomes and (b) lysosomes were detected using specific dyes. The formation of autophagosomes and lysosomes increased after MG treatment compared to the vehicle. Data are presented as the mean±SD of three independent experiments in triplicate (** p<0.01).

Autophagy may play an important role in treatment response of cancer. We further checked whether or not MG can influence activation of the autophagic pathway. Autophagic vacuoles and autophagic flux were measured with a CYTO-ID^®^ Autophagy Detection Kit. Fluorescent detection was evaluated in the vehicle control and MG-treated HepJ5 and Mahlavu cells at 24 h. Treatment of HepJ5 and Mahlavu cells with MG caused induction of autophagosome formation (a 2-fold increase in the fluorescence intensity) compared to vehicle-treated cells (Fig 5A). In addition, we further detected lysosome formation with a LYSO-ID® Green Detection Kit. Cells were incubated with LYSO-ID® Green dye after incubation with MG for 24 h. MG-treated HepJ5 and Mahlavu cells displayed a 4-fold greater fluorescence intensity than vehicle-treated HepJ5 and Mahlavu cells (Fig 5B). These results suggested that MG treatment induces autophagosome and lysosome formation in HCC cells.

### 3.5. MG affects expressions of proteins associated with autophagy

**Fig 6:**
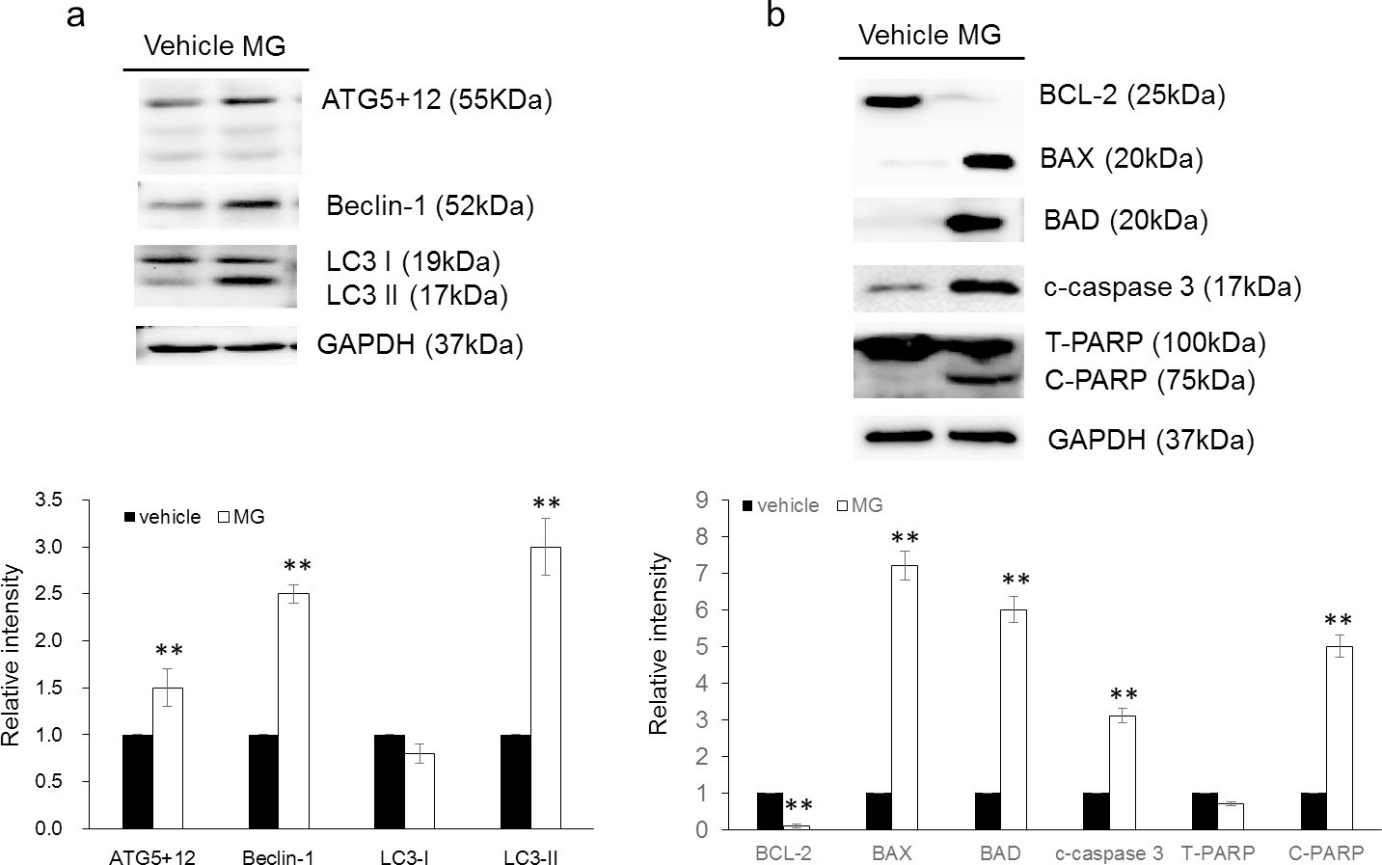
Methyl Gallate (MG) treatment causes changes in autophagy- and apoptosis-related proteins. HepJ5 cells were treated with 40 μg/ml MG or vehicle for 48h. **a**. Levels of autophagy-related proteins (ATG5, ATG12, Beclin-1, and LC3) were checked by western blot. Amounts of ATG5, ATG5+12, and Beclin-1 were similar between MG-treated and vehicle-treated samples. The ratio of LC3-II/LC I was high in the MG-treated sample. **b.** Levels of cell apoptosis-related proteins (Bcl-2, Bax, Bad, c-caspase 3 and c-PARP) were checked. MG-treated cells showed decreased Bcl-2 (antiapoptotic), increased Bax and Bad proteins (proapoptotic), and increased cleavage of caspase 3 (c-caspase3) and PARP (c-PARP). All experiments were repeated at least three times independently (** *p*<0.01).

Western blot analysis was used to confirm that MG induced autophagic signals in HepJ5 cells. Expressions of ATG5/12, LC3-I, LC3-II, and Beclin-1 proteins were analyzed, all of which contribute to activation of downstream autophagy components. MG treatment induced upregulation of ATG 5+12 and Beclin-1 and the conversion of LC3-I to LC3-II, which confirmed induction of autophagy in HepJ5 (Fig 6A).

### 3.6. MG affects expressions of proteins associated with apoptosis

We next examined the effect of MG on caspase-dependent apoptosis in HepJ5 cells. A western blot analysis demonstrated that treatment of HepJ5 cells with MG resulted in upregulation of c-PARP and c-caspase3 (Fig 6B). Decreased antiapoptotic (Bcl-2) members and increased proapoptotic (Bax and Bad) members suggested that MG induced caspase-dependent apoptosis in HepJ5 cells.

### 3.7. Blocking autophagy enhances the apoptotic effect of MG in HCC

**Fig 7:**
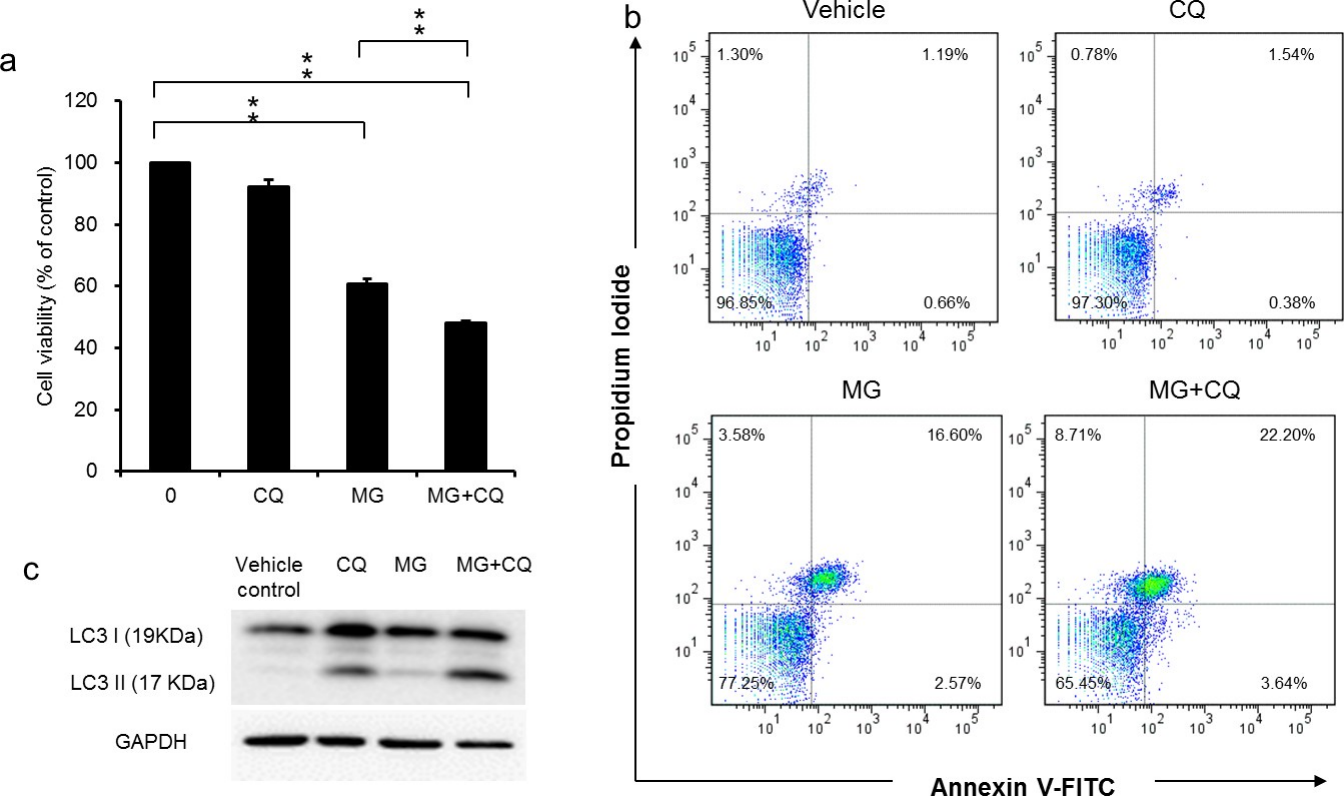
Blocking autophagy enhances MG-induced cytotoxicity in HepJ5 cells. The cell viability in cells treated with MG for 24 h in the presence and absence of CQ for 16 h was determined using SRB assay (a). Apoptotic cells were quantified using Annexin V-FITC/PI staining and FACS analysis after 40 μg/ml MG treatment for 48h in the presence and absence of CQ for 16 h (b). The blockage of autophagy flux was confirmed by detecting the accumulation of LC3-II using western blot (c). Data are presented as the mean±SD of three independent experiments in triplicate, ** p<0.01).

To determine the role of autophagy in regulating MG-induced cell death in HCC, CQ (chloroquine, a lysosomal inhibitor) was used in MG-treated HepJ5 cells. It was found that combinatorial treatment of CQ significantly increased MG-induced cytotoxicity in HCC. As shown in Fig 7A, the cell viability was decreased to 60.8% and 48.0% following 40 μg/ml MG and MG plus 10 μM CQ treatment, respectively. However, CQ treatment only did not affect the cell viability. Furthermore, CQ markedly enhanced MG‐induced apoptosis in HCC (Fig 7B). The percentage of apoptotic cells was 25.8% after combination treatment of MG and CQ compare with 19.2% of the cells treated with MG for 48h in HepJ5. In addition to autophagy initiation, the accumulation of LC3-II may result from impaired autophagic flux. Therefore, the blockage of autophagy flux was confirmed by detecting the accumulation of LC3-II. The data showed that LC3-II expression was dramatically increased in CQ-treated cells and in combinatorial treatment with MG (Fig 7C). Taken together, these results suggested that MG induced protective autophagy and cytotoxicity in HCC, and blocking autophagy flux increased MG-induced cell death in HCC.

## 4. Discussion

HCC is a highly prevalent cancer, and there is a significant geographical epidemiology, as 80% of new cases occur in developing countries, such as southern Africa and Asia [40]. The long-term survival rate of advanced HCC has remained poor in the past several decades [41]. Finding powerful agents that work effectively and efficiently through various antitumor mechanisms is urgently needed. Plants synthesize a wide range of natural compounds, and some of the metabolites act as antioxidant and antitumor drugs because of their cytotoxic effects toward malignant cells [25, 42]. Some natural compounds, such as curcumin and β-glucans, suppress cell proliferation and induce apoptosis in HCC cells [43–45]. MG is a natural phenolic agent from plants and a derivative of gallic acid [46]. The current study focused on examining the anticancer effects of MG on HCC.

MG exhibits multiple biological properties such as antioxidant, anti-inflammatory, antimicrobial, antitumor activities [34, 46, 47]. In the inflammatory response, mitochondrial ROS support a balance between mitogen-activated protein kinases and cytokines [48]. Furthermore, immunotherapy with MG enhanced the anticancer effect of cisplatin in lymphoma treatment [26]. Our data indicate the antitumor effects of MG via ROS-dependent cell death. Cancer cells generate a variety of endogenous ROS, but they are vulnerable to increased and prolonged exposure to ROS. Many chemotherapeutics increase intracellular ROS levels in order to induce the apoptosis of cancer cells. ROS are chemically reactive molecules that increase during environmental stress. ROS, including peroxides and superoxide, are generated as byproducts of mitochondrial metabolism [49]. In addition, oxidative stress caused by excess ROS leads to loss of the mitochondrial membrane potential and induces cytochrome C release [50]. Our results indicated that the levels of oxidation and superoxide production dramatically increased after MG exposure (Fig 3) and then induced apoptosis and autophagy of MG-treated HCC cells.

DNA damage and mitochondrial dysfunction mediate apoptosis and autophagy. ROS levels play a role in cell death through activating different signaling pathways, including AKT/mammalian target of rapamycin [51, 52]. Excess mitochondrial ROS production modulates progressive autophagy [53, 54]. Redox homeostasis can determine the fate of cancer cells through various signaling pathways, including apoptosis, autophagy, and cell cycle arrest [55]. MG-caused tumor cell death, including by apoptosis, is dependent on ROS production. MG-triggered ROS and superoxide were markedly reversed by aminoguanidine hemisulfate (AGH), a well-known antioxidant (Fig 4). In our study, the anticancer effect induced by MG in HCC cells in vitro were additionally demonstrated in the experimental zebrafish xenograft model.

Apoptosis is the most comprehensive form of programmed cell death [56]. The caspase pathway plays a role in intrinsic and extrinsic apoptosis [57]. The intrinsic apoptotic pathway is modulated by the Bcl-2 family, which involves proapoptotic proteins, including Bad, Bak, and Bax, as well as the antiapoptotic proteins, Bcl-2 and Bcl-XL [58]. Increased c-PARP and c-caspase-3 and decreased expression of Bcl-2 were demonstrated in our study. Increased Bax and Bad expressions indicated that MG triggered mitochondrial-specific apoptosis in HCC cells. We observed the early and late stages of apoptosis, and even necrosis, in HCC cells treated with MG.

Autophagy is initiated to produce intracellular energy and nutrients, and cells self-eat their unfolded proteins and organelles to maintain homeostasis [59, 60]. Recent reports linked autophagy to failure of clinical cancer treatments, including chemo- and radio-resistance [61]. Under stressful conditions, cancer cells induce autophagy, thus promoting cell survival in a nutrition-deprived situation. Activation of the autophagic pathway acts in both protective and inhibitory roles in cancer progression [62]. Autophagy-related 5 (ATG5) is a key protein involved in autophagic vesicles. ATG5 is necessary for LC3-I to form LC3-II (LC3-phosphatidylethanolamine conjugate) [63]. During progressive autophagy, LC-3 is cleaved to LC3-II on membranes of autophagosomes. Beclin-1 is a key molecule for autophagosome formation, and is a vital component of the class III phosphatidylinositol 3 kinase complex [64]. Our results indicated MG treatment produced increases in Beclin-1 and ATG5 + ATG12 expressions, and the conversion of LC3-I to LC3-II (Fig 6), which is consistent with MG-treated cells inducing autophagosome and lysosome formation. In our study, elevated ROS levels, cell apoptosis and progressive autophagy phenomena occurred in MG treated-HCC cells. And blocking autophagy increased MG-induced cytotoxicity.

Our studies demonstrated that MG inhibits human HCC cells via apoptosis. Multiple hypothetical pathways were discussed before by Chen et al. [65] who showed GSH depletion, caspase and MAPK activation, and upregulation of p53, Bax, Fas, and Fas-L expressions in leukemia cells after propyl gallate (PG), the MG-related gallate, treatment. Interestingly, MG’s induction of GSH depletion and cell death in leukemia cells did not result from increasing ROS levels. MG inhibited the nuclear translocation of Nrf-2, sequenced by c-GCS downregulation, which may ultimately result in GSH depletion in MG-treated leukemia cells. MG may play a different role in ROS levels, and the cell response depends on different MG concentrations, treatment durations, or cell types. In conclusion, MG effectively inhibited HCC cells both in vivo and in vitro. Treatment of HCC cells with MG increased intracellular ROS and superoxide levels, upregulated ATG5-ATG12 complex and Beclin-1, and converted LC3-I to LC3-II, all of which are essential to the induction of autophagy of HCC cells. And our results revealed that autophagy activation was a protective response against MG-induced cell death in HCC. These findings suggest that MG might be a promising therapeutic agent against HCC development.
